# Early Warning Surveillance for SARS-CoV-2 Omicron Variants, United Kingdom, November 2021–September 2022

**DOI:** 10.3201/eid2901.221293

**Published:** 2023-01

**Authors:** Sarah Foulkes, Edward J.M. Monk, Dominic Sparkes, Nipunadi Hettiarachchi, Iain D. Milligan, Katie Munro, Andrew Taylor-Kerr, Naomi Platt, Anna Howells, Jerry Ye Aung Kyaw, Enemona Adaji, Eileen Gallagher, Jameel Khawam, Edgar Wellington, Lesley Price, David Crossman, Chris Norman, Elen de Lacy, Lisa Cromey, Diane Corrigan, Angie Lackenby, Paola Barbero, Busayo Elegunde, Maria Zambon, Meera A. Chand, Colin S. Brown, Jasmin Islam, Ana Atti, Susan Hopkins, Victoria J. Hall, Michelle J. Cole, the SIREN Study Group

**Affiliations:** UK Health Security Agency, London, UK (S. Foulkes, E.J.M. Monk, D. Sparkes, N. Hettiarachchi, I.D. Milligan, K. Munro, A. Taylor-Kerr, N. Platt, A. Howells, J. Ye Aung Kyaw, E. Adaji, E. Gallagher, J. Khawam, E. Wellington, A. Lackenby, P. Barbero, B. Elegunde, M. Zambon, M.A. Chand, C.S. Brown, J. Islam, A. Atti, S. Hopkins, V. Hall, M.J. Cole);; Royal Free London NHS Foundation Trust, London (I.D. Milligan);; Glasgow Caledonian University, Glasgow, Scotland, UK (L. Price);; Public Health Scotland Glasgow Office, Glasgow (L. Price);; University of St. Andrews, St. Andrews, Scotland, UK (D. Crossman);; Health and Care Research Wales, Cardiff, Wales, UK (C. Norman);; Public Health Wales, Cardiff (E. de Lacy); Public Health Agency Northern Ireland, Belfast, Northern Ireland, UK (L. Cromey, D. Corrigan);; Guy’s and St. Thomas’ NHS Foundation Trust, London (M.A. Chand); Royal Free Hospital, London (C.S. Brown);; National Institute for Health Research Health Protection Research Unit in Healthcare Associated Infections and Antimicrobial Resistance, London (S. Hopkins, V.J. Hall)

**Keywords:** COVID-19, respiratory infections, severe acute respiratory syndrome coronavirus 2, SARS-CoV-2, SARS, coronavirus disease, zoonoses, viruses, coronavirus, Omicron variant, BA.1, BA.2, BA.4, BA.5, SIREN study, United Kingdom

## Abstract

Since June 2020, the SARS-CoV-2 Immunity and Reinfection Evaluation (SIREN) study has conducted routine PCR testing in UK healthcare workers and sequenced PCR-positive samples. SIREN detected increases in infections and reinfections and detected Omicron subvariant emergence contemporaneous with national surveillance. SIREN methodology can be used for variant surveillance.

Since June 2020, the SARS-CoV-2 Immunity and Reinfection Evaluation (SIREN) Study has detected and investigated SARS-CoV-2 reinfections in the United Kingdom; after vaccine rollout, SIREN was adapted to monitor vaccine effectiveness (*1*–*5*). As the United Kingdom, like other countries, adapts to the postacute phase of the pandemic and reduced testing availability (*6*,*7*), SIREN has an ongoing function in national surveillance. SIREN informs the UK pandemic response by real-time monitoring of emerging variants and determining national rates of primary infection and reinfection. We describe SIREN’s surveillance strategy and characterize emergence of Omicron subvariants during successive waves within the study.

## The Study

SIREN is a large, multicenter, prospective cohort study of >44,000 UK healthcare workers from 135 secondary care health organizations. SIREN is led by the UK Health Security Agency in collaboration with Public Health Wales, Public Health Scotland, and the Public Health Agency Northern Ireland (*1*). Participants were initially followed for 12 months and had an option to extend to 24 months. Participants completed an initial enrollment survey regarding demographic and occupational data, then completed follow-up surveys every other week regarding symptoms, vaccination status, and occupational, household, and community SARS-CoV-2 exposures. Participants underwent PCR testing every 2 weeks and serologic testing monthly for the first 12 months, then had quarterly serologic testing. We confirmed vaccination status through linkage to personal identifiable information in national vaccination registries (*1*). This study was approved the Berkshire Research Ethics Committee (approval no. IRAS ID 284460, REC reference no. 20/SC/0230) on May 22, 2020; the vaccine amendment was approved on January 12, 2021 (study registration no. ISRCTN11041050).

SIREN samples were processed according to local protocols. Data from sites were supplied through the national laboratory reporting systems and obtained through linkage to personal identifiers. SARS-CoV-2 testing records for all participants, including symptomatic PCR testing outside SIREN’s protocol, were stored in the SIREN database (*1*).

When RNA load was sufficient, local SIREN teams referred PCR-positive samples for sequencing (*1*). An additional self-swab kit for centralized PCR testing and sequencing at the national reference laboratory in London was initially mailed to participants who had a new infection and a history of SARS-CoV-2 primary infection or COVID-19 vaccination. Since spring 2021, participants with a positive PCR, irrespective of previous infection and vaccination status, were mailed an additional self-swab kit when available, which maximized the opportunity to improve sequencing yields.

We defined primary infection as a PCR-positive test from a participant without laboratory evidence of prior infection, such as a positive PCR test or antibody positivity before first vaccination (*1*). We defined reinfection as 2 PCR-positive tests separated by >90 days; or before a participant was vaccinated, a PCR-positive test >28 days after first SARS-CoV-2 IgG detection.

**Figure 1:**
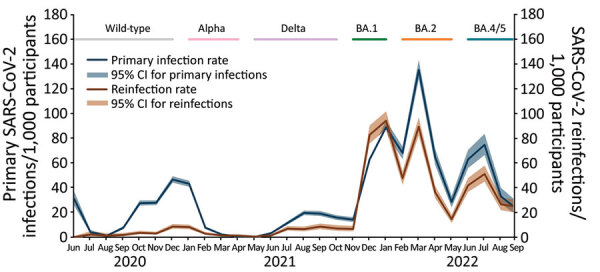
Rate of SARS-CoV-2 infections and reinfections detected through SIREN early warning system, United Kingdom, June 2020–September 2022. The SIREN study tested healthcare workers every 2 weeks via PCR and sequenced PCR-positive samples. We considered primary infection as infections among participants without prior infection and reinfections as infections among persons with prior infection. SIREN, SARS-CoV-2 Immunity and Reinfection.

Since June 2020, SIREN monthly infection rates show 6 distinct infection waves (Figure 1). These waves corresponded with wild-type SARS-CoV-2 and subsequent emergence of Alpha variant; Delta variant; and Omicron BA.1, BA.2, and BA.4/BA.5 subvariants. Those waves are consistent with national surveillance trends (*8*).

Infection rates during Omicron BA.1 and BA.2 subvariant dominance surpassed those observed in any previous wave (Figure 1). This dominance was most apparent for reinfection rates, which exceeded primary infection rates for the first time in December 2021 and peaked at 94.2 reinfections/1,000 participants tested in January 2022 (Figure 1). The study cohort was well characterized and highly vaccinated and had high rates of prior infections. Thus, SIREN data suggest infection-acquired and vaccine-acquired immunity were less protective against Omicron BA.1 subvariant infection.

Since May 2022, we have detected a sixth wave of SARS-CoV-2 infections in the SIREN cohort, and rates continue to increase across the United Kingdom. These findings coincide with emergence of newer Omicron BA.4 and BA.5 subvariants (*8*). In contrast to the first wave of Omicron BA.1 variant infections, rates of reinfections remain considerably lower than primary infections, likely because protection improved after BA.1 and BA.2 infections and vaccination.

During March 3, 2020–September 30, 2022, SIREN recorded 18,319 participant infection episodes, of which 5,261 (28.7%) had valid sequence and infection data available, comprising 4,085 primary infections and 1,1176 reinfections. Our sequence yield was comparable to other studies (*8*,*9*), but we continue to expand data linkage and sample flows to improve data completeness.

**Figure 2:**
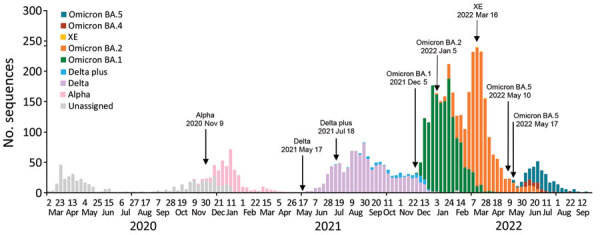
Number of sequenced samples by variant per week in SIREN early warning system, United Kingdom, March 2020–June 2022. The SIREN study tested healthcare workers every 2 weeks via PCR and sequenced PCR-positive samples. We have plotted all samples successfully sequenced and assigned a variant call or unclassified lineage. Dates of detection are noted for each variant. Of note, among >44,000 UK healthcare workers from 135 secondary care health organizations, we detected 521 cases of unclassified variants, 323 cases of Alpha, 1,042 cases of Delta, 83 cases of Delta plus, 1,487 cases of Omicron BA.1, 1,514 cases of Omicron BA.2, 4 cases of XE, 51 cases of Omicron BA.4, and 236 cases of Omicron BA.5. SIREN, SARS-CoV-2 Immunity and Reinfection.

SIREN sequencing identified several SARS-CoV-2 variants (Figure 2). Before Omicron subvariants emerged, 1,969 sequences with linked infection episode data were available: 521 unclassified/wild-type, 323 Alpha variant, 1,042 Delta variant, and 83 Delta plus subvariant. Among these sequences, 8.3% (164/1,969) were reinfections and 91.7% (1,805/1,969) were primary infections.

The Omicron BA.1 subvariant was detected in SIREN on December 5, 2021, and 1,487 cases were detected by September 30, 2022; of those, 31.0% were reinfections. Omicron BA.2 was detected during January 5–September 30, 2022, and caused 1,514 cases, 27.3% of which were reinfections. Since March 2022, other Omicron subvariants have been identified within SIREN. The XE BA.1/BA.2 recombinant was detected from 4 primary infections on March 16, 2022. The BA.4 subvariant (25 primary infections and 26 reinfections) was detected on May 10, 2022, and the BA.5 subvariant (126 primary infections and 110 reinfections) was detected on May 17, 2022. Except for 4 XE cases, initial detection dates for Omicron subvariants within SIREN were an average of 16 days later than the Office for National Statistics (ONS) (*10*) and 27 days later than national surveillance (*8*). ONS detected BA.1 on November 29, 2021, national surveillance detected it November 3, 2021; ONS detected BA.2 on January 3, 2022, national surveillance detected it December 19, 2021; ONS detected BA.4 on April 4, 2022, national surveillance detected it April 12, 2022; and ONS detected BA.5 April 18, 2022, national surveillance detected it April 17, 2022 (*8*,*10*). We consider SIREN dates comparable with national surveillance data, which also contains sequence data from travelers and focused variant detection exercises.

## Conclusions

Established early in the COVID-19 pandemic, SIREN has monitored infection trends and emerging variants for >2 years, directly informing the United Kingdom’s national response (*2*–*4*), and contributing to Variant Technical Group briefings and government reports (*8*,*11*). After 4 successive variant waves with similar reinfection profiles, SIREN data showed that Omicron BA.1 and BA.2 subvariants emerged and caused a rapid rise in primary infection and reinfection rates among SIREN participants, regardless of vaccination status. Each subsequent Omicron subvariant was detected within a similar timeframe to national data (*8*), authenticating SIREN’s use as a robust and representative surveillance tool.

The SIREN study population represents a highly exposed group who also have contact with vulnerable patients. In the absence of universal symptomatic PCR testing, SIREN provides a sustainable, focused, objective-driven sentinel surveillance platform and access to key epidemiologic variables, such as symptom severity. Although our cohort of predominantly healthy, working age, highly vaccinated adults are not representative of the general population, our study complements other national surveillance programs that target community and older populations (*10*,*12*). SIREN continues to improve its surveillance across a sentinel healthcare worker network in 135 national health organizations and support timely detection of infection trends and emerging variants, keeping pace with other surveillance tools.

After a sixth wave of UK infections, concerns have grown around winter healthcare pressures combined with high rates of influenza observed in the Southern Hemisphere (*13*). Ongoing effective COVID-19 surveillance integrated with influenza and other respiratory pathogen surveillance is essential and achievable through SIREN’s sentinel nature (*7*,*14*). SIREN will continue testing through March 31, 2023, and will continue to be a key asset in the UK surveillance strategy (*7*,*14*). The findings from SIREN can inform other countries’ transitions from comprehensive surveillance to sentinel surveillance of key populations, such as healthcare workers.

## References

[1] Wallace S, Hall V, Charlett A, Kirwan PD, Cole M, Gillson N, et al. Impact of prior SARS-CoV-2 infection and COVID-19 vaccination on the subsequent incidence of COVID-19: a multicentre prospective cohort study among UK healthcare workers - the SIREN (Sarscov2 Immunity & REinfection EvaluatioN) study protocol. BMJ Open. 2022;12:e054336. 10.1136/bmjopen-2021-054336 35768083 PMC9240450

[2] Hall VJ, Foulkes S, Charlett A, Atti A, Monk EJM, Simmons R, et al.; SIREN Study Group. SARS-CoV-2 infection rates of antibody-positive compared with antibody-negative health-care workers in England: a large, multicentre, prospective cohort study (SIREN). Lancet. 2021;397:1459–69. 10.1016/S0140-6736(21)00675-9 33844963 PMC8040523

[3] Hall VJ, Foulkes S, Saei A, Andrews N, Oguti B, Charlett A, et al.; SIREN Study Group. COVID-19 vaccine coverage in health-care workers in England and effectiveness of BNT162b2 mRNA vaccine against infection (SIREN): a prospective, multicentre, cohort study. Lancet. 2021;397:1725–35. 10.1016/S0140-6736(21)00790-X 33901423 PMC8064668

[4] Hall V, Foulkes S, Insalata F, Kirwan P, Saei A, Atti A, et al.; SIREN Study Group. Protection against SARS-CoV-2 after Covid-19 vaccination and previous infection. N Engl J Med. 2022;386:1207–20. 10.1056/NEJMoa2118691 35172051 PMC8908850

[5] Atti A, Ferrari M, Castillo-Olivares J, Monk EJM, Gopal R, Patel M, et al. Serological profile of first SARS-CoV-2 reinfection cases detected within the SIREN study. J Infect. 2022;84:248–88. 10.1016/j.jinf.2021.09.019 34600935 PMC8482544

[6] Cabinet Office. COVID-19 response: living with COVID-19. 2022 May 06 [cited 2022 Jul 14]. https://www.gov.uk/government/publications/covid-19-response-living-with-covid-19

[7] Suk JE, Pharris A, Beauté J, Colzani E, Needham H, Kinsman J, et al. Public health considerations for transitioning beyond the acute phase of the COVID-19 pandemic in the EU/EEA. Euro Surveill. 2022;27:2200155. 10.2807/1560-7917.ES.2022.27.17.2200155 35485272 PMC9052765

[8] UK Health Security Agency. SARS-CoV-2 variants of concern and variants under investigation in England; technical briefing 42; 20 May 2022 [cited 2022 Jul 14]. https://assets.publishing.service.gov.uk/government/uploads/system/uploads/attachment_data/file/1103534/Technical-Briefing-42-20May2022.pdf

[9] European Centre for Disease Prevention and Control. Country overview report [cited 2022 Jul 22]. https://www.ecdc.europa.eu/en/covid-19/country-overviews

[10] Office for National Statistics. Coronavirus (COVID-19) infection survey: technical data [cited 2022 Jul 22]. https://www.ons.gov.uk/peoplepopulationandcommunity/healthandsocialcare/conditionsanddiseases/datasets/covid19infectionsurveytechnicaldata

[11] UK Health Security Agency. SIREN study. 2022 Jun 21 [cited 2022 Jul 14]. https://www.gov.uk/guidance/siren-study

[12] Krutikov M, Palmer T, Donaldson A, Lorencatto F, Forbes G, Copas A, et al. Study Protocol: Understanding SARS-Cov-2 infection, immunity and its duration in care home residents and staff in England (VIVALDI). Wellcome Open Res. 2021;5:232. 10.12688/wellcomeopenres.16193.2 33564722 PMC7851710

[13] Australian Government Department of Health. Australian influenza surveillance report no. 6; 2022 Jun 19 [cited 2022 Jul 14]. https://www1.health.gov.au/internet/main/publishing.nsf/Content/828055131E7175CCCA25886B001C60FC/$File/flu-06-2022.pdf

[14] European Centre for Disease Prevention and Control. COVID-19 surveillance guidance. 2021 Oct 1 [cited 2022 Jul 14]. https://www.ecdc.europa.eu/sites/default/files/documents/COVID-19-surveillance-guidance.pdf

